# A rare case of delayed anaplasma phagocytophilum-induced pancytopenia: A diagnostic conundrum

**DOI:** 10.1016/j.amsu.2022.103366

**Published:** 2022-02-11

**Authors:** David Song, Talal Almas, Mohamed Abdelghffar, Samkit Jain, Harinivaas Shanmugavel Geetha, Vaibhav Shah, Vikneswaran Raj Nagarajan, Norah Alshareef, Varman Gunasaegaram, Keesha Ravintharan, Sze Teng Tan, Arun John

**Affiliations:** a Icahn School of Medicine at Mount Sinai - Elmhurst Hospital Center, Elmhurst, NY, USA; b RCSI University of Medicine and Health Sciences, Dublin, Ireland; c Saint Vincent Hospital, Worcester, MA, USA; d University Hospital, National University of Ireland—Galway, Galway, Ireland; e Kuala Lumpur Hospital, KL, Malaysia

**Keywords:** Anaplasmosis, Pancytopenia, Ticks

## Abstract

**Introduction:**

Human granulocytic anaplasmosis (HGA) is a potentially fatal tick-borne disease caused by the obligate intracellular bacterium Anaplasma phagocytophilum. It is most commonly found in the Northeastern and Midwestern parts of the United States especially during spring and summer months. The clinical picture of anaplasmosis is varied ranging from common symptoms such as fever, headache and myalgia to rarer presentations such as pancytopenia.

**Case presentation:**

We present a case of a 62 year old male who presented with watery diarrhea, fever, and pancytopenia. Although there is a broad differential for pancytopenia, a thorough history provides clues regarding the diagnosis. In our patient, a recent history of camping in Upstate New York was suggestive of an infectious etiology from a tick borne illness.

**Clinical discussion:**

A tick-borne panel guided us to identify the diagnosis of HGA. Although the exact underlying pathogenesis of tick-borne illnesses leading to pancytopenia is still unknown, the pancytopenia is postulated to be due to a multi-nodal mechanism involving immune and non immune platelet destruction, global bone marrow suppression, hemophagocytic lymphohistiocytosis and myelosuppressive chemokines release.

**Conclusion:**

We hope that this case report elucidates the importance of obtaining a meticulous history in guiding clinicians towards prompt diagnosis, even in instances where there may be an evolving clinical picture.

## Highlights

• Human granulocytic anaplasmosis (HGA) is a potentially fatal tick-borne disease caused by the obligate intracellular bacterium *Anaplasma phagocytophilum*.
• A prompt diagnosis and management will facilitate an early recovery and prevent any fatal complications such as pancytopenia.
• HGA causing pancytopenia is likely due to a multi-modal mechanism involving immune and non-immune platelet destruction, global bone marrow suppression, hemophagocytic lymphohistiocytosis, and myelosuppressive chemokines release.

## 1 Introduction

Human granulocytic anaplasmosis (HGA) is a potentially fatal tick-borne disease caused by the obligate intracellular bacterium Anaplasma phagocytophilum, which has a tropism for neutrophils. It typically occurs in the warm spring and summer months and is most commonly found in the Northeastern and Midwestern parts of the United States. Although the case fatality rate of anaplasmosis has remained low (<1%) according to Center for Disease Control and Prevention (CDC) reports, there should be a high suspicion for tick borne illnesses as it initially presents with non-specific symptoms and the absence of any unique pathological feature that helps in the early diagnosis [1]. It is transmitted by the Ixodes Scapularis tick (deer tick), which is also the known vector for transmitting lymes and babesia. It is also carried by other tick species in Europe and Asia. In New York State, it is predominantly found along the Hudson River Valley. Clinical symptoms of HGA include fever, headache, myalgia, neutropenia and thrombocytopenia [2]. In rare cases, it can present as pancytopenia. Signs and symptoms may develop up to two weeks after the initial tick bite. Similar to other tick borne illnesses, first line treatment for HGA is doxycycline. Ten-day course of doxycycline can be started as soon as there is high suspicion with or without a confirmed diagnosis by PCR testing. Cell line recovery and resolution of symptoms is seen within days of starting antibiotics. In addition, this work has been reported in accordance with SCARE [3].

## 2 Case presentation

Patient is a 62 year old male with a past medical history of hypertension presented to the emergency department (ED) with 4 days of watery, non-bloody diarrhea occurring throughout the day with concurrent diaphoresis, chills, and high fever ranging from 102 to 105 F with minimal improvement on acetaminophen. He reported a significant decrease in appetite and oral intake. He had a recent trip to upstate New York and returned about 2 weeks ago. During this trip, he noticed numerous mosquito bites, but denied having any rashes, tick bites, sick contacts or any travel abroad. On exam, he was found to be diaphoretic, tachycardic and febrile with a temperature of 102.9F. The abdominal exam revealed normal bowel sounds in all four abdominal quadrants, was nondistended, soft and nontender to palpation. No hepatosplenomegaly was present.

On admission the labs were notable for white blood cell count 3.08 × 10^3^/mcL (normal: 4.8–10.80 × 10^3^/mcL), which reduced to 0.93 × 10^3^/mcL during the hospitalization day 2, hemoglobin 15.5 g/dL (normal: 14.0–18.0 g/dL) initially but 11.1 g/dL on hospitalization day 2, platelet count 27,000/mcL (normal: 150-450 × 10^3^/mcL), fibrinogen 410 mg/dL (normal: 200–393 mg/dL), lactate dehydrogenase (LDH) 511 U/L (normal: 135–225 U/L), and D-Dimer: 6,172 ng/mL DDU (normal: 0–243 ng/mL DDU), aspartate aminotransferase 76 U/L (normal: 5–40 U/L) and rest of the lab including haptoglobin, human immunodeficiency virus (HIV), hepatitis panel and electrolytes were within normal range. Hematology was also consulted for pancytopenia and direct antiglobulin tests IgG level and C3 level were negative, flow cytometry studies were unremarkable, and no schistocytes seen on peripheral smear. The common causes of secondary immune thrombocytopenic purpura (ITP) were ruled out using the lab results. The patient was negative for HIV and HEP C, which are the two predominant viral causes of secondary ITP.

The fibrinogen levels were normal and peripheral smear did not show microangiopathic pathology which outlawed the possibility of disseminated intravascular coagulation (DIC).

**Fig. 1:**
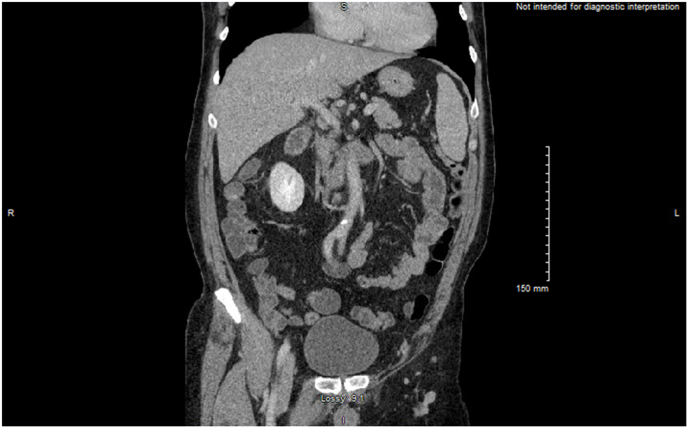
Computed Tomography Angiography of Abdomen & Pelvis with contrast displaying intraluminal fluid enhancement in parts of small bowel suggestive of viral enterocolitis (Coronal view).

**Fig. 2:**
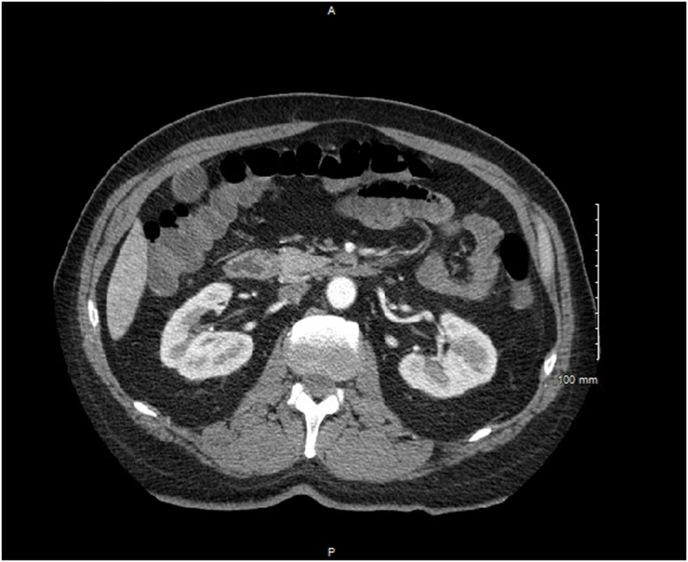
Computed Tomography Angiography of Abdomen & Pelvis with contrast displaying intraluminal fluid enhancement in the colon suggestive of viral enterocolitis. No bowel wall thickening or bowel edema reflective of inflammatory changes was noted. (Axial View).

Chest radiography was negative for consolidation, or infiltrates and the computed tomography (CT) Pulmonary angiography was negative for pulmonary embolism. The CT abdomen & pelvis with contrast showed an incidental 11mm cystic lesion adjacent to the pancreatic body and primarily emphasized findings suggesting viral enterocolitis (Fig. 1, Fig. 2).

The patient received multiple units of platelet transfusion in the setting of febrile thrombocytopenia less than 20,000/mcL. The patient was treated with empiric antibiotics given the concern for infection with vancomycin 1000mg daily, cefepime 1g every 12 hours and metronidazole 500mg every 8 hours to cover anaerobes given CT findings. Full infectious workup including blood cultures, stool culture, clostridium difficile antigen, ova and parasite tests were all negative. Tick borne infectious disease serologies were unremarkable for babesia, rocky mountain spotted fever, ehrlichia or lyme antibodies. Ultimately, the patient was found to have anaplasma phagocytophilum antibodies positive and a diagnosis of HGA was made. The antibiotics were stopped and were switched to doxycycline 100mg every 12 hours with planned for ten days. The patient had a rapid clinical improvement and the pancytopenia improved. The patient was discharged home to complete the ten day course of doxycycline and was followed up in the outpatient clinic. At the outpatient clinic, the patient continued to show improvement of his symptoms and labs showed resolution of pancytopenia. The predominant challenge in this case was figuring out the cause of the pancytopenia in this patient. It was crucial for the clinician to dig deep and have a detailed history by covering salient points, giving the treating clinician a more holistic picture in order to ascertain the cause of pancytopenia. Furthermore, the investigative workup was initially significant only for thrombocytopenia, lending to the possibility of conversion to pancytopenia after one week.

## 3 Discussion

Pancytopenia is a common hematologic manifestation seen in clinical practice with decrease in all three cell lines [[4], [5]]. It generally has a broad differential including malignancy, autoimmune disorders, viral infections, drug side effects, and nutritional disorders. Pancytopenia presents with a variety of clinical features such as fever, weight loss, dyspnea, and generalized weakness. History often plays a key role in determining the etiology of pancytopenia[5]. Salient features in the history such as camping, trekking, and hiking in the northeastern states especially during spring and summer are highly suggestive of tick borne diseases and helps us narrow down the differential diagnosis. An early diagnosis becomes vital in the management of pancytopenias especially the tick borne disease as they are easily treatable. The main practical takeaway from this case would be to reiterate the importance of meticulous history taking and considering the different possibility of differentials from the start, even in instances where there may be an involving investigative picture. This ensures that differentials can be ruled out with investigations and the next can be considered leading to early diagnosis and early treatment, lending to better prognostic outcomes.

There has yet to be any conclusive data frequency of HGA induced pancytopenias as the presentation of this is rare. Various reports have shown that individual cytopenias such as leukopenia and thrombocytopenia occur in up to 80% of cases with thrombocytopenia being the more common presentation among the two [[4], [5]]. The mainstay of treatment of HGA are with antibiotics, namely Doxycycline. Rifampin can be used if there's evidence of intolerance or allergies to tetracyclines however caution is advised as the efficacy of rifampin is still undetermined[5]. In patients with severe pancytopenia, the use of granulocyte colony stimulating factors such as Filgrastim have been raised however more detailed research into the use of G-CSF and its efficacy in improving neutropenias in tick-borne illnesses is required. The question of pl.

Anaplasma infection is one of the important tick borne diseases causing pancytopenia, which if left untreated, can prove to be fatal [[4], [5]]. Although the exact underlying pathogenesis of anaplasma infection causing pancytopenia is still unknown, there are several well mechanisms that have been postulated. One of the most accepted mechanisms is the release of myelosuppressive chemokines due to anaplasma infection. Studies revealed that in infected cells, there is an upregulation of chemokines such as MCP-1, MIP-1 alpha and beta, and IL-8 [5]. These chemokines have been demonstrated to decrease the proliferation and differentiation of myeloid progenitor cells. Meanwhile, classic proinflammatory cytokines such as IL-1,IL-6 and TNF-alpha were found to be downregulated in the infected cells. This evidence supports that anaplasma infection causes cytopenias through myelosuppressive chemokines [2,4]. One of the features of anaplasma infection that supports this mechanism is the increase in the number of hematopoietic stem cells in the spleen [6]. Splenomegaly was observed due to lymphoid hyperplasia and erythropoiesis secondary to bone marrow suppression further demonstrating the endotoxin mediated effects on the bone marrow and spleen [6]. However, anaplasma has also been shown to affect the bone marrow progenitor cells by affecting the signalling pathways. Molecular analysis into the mechanism of myelosuppression revealed that anaplasma infection leads to decreased signalling of the CXCL12/CXCR4 pathway which plays a crucial role in hematopoietic stem cell and progenitor cell maturation and mobilization. These evidence suggest that anaplasma affects hematopoiesis at the molecular level making reversal of these cytopenias crucial [7].

One of the mechanisms that has been described several times in the literature is Hemophagocytic lymphohistiocytosis (HLH). HGA causing HLH is first thought to have been described by Dumler et al., in 2007 where 29 patients had HGA and among case fatalities who were autopsied, hemophagocytic macrophages were found in the spleen among other organs [[7], [8]]. Yi et al. has also described the incidence of HLH in patients infected with Anaplasma further supporting the evidence that HLH is also one of the mechanisms by which Anaplasma causes cytopenias in patients [8,9]. Anaplasma infection has also been proven to preferentially infect mature granulocytes. Studies have demonstrated that anaplasma does not directly infect the progenitor cells in the bone marrow. Bayard et al. has demonstrated that it has a predilection to infect and destroy peripheral mature granulocytes [[10], [11]].

All these evidences obtained from literatures suggest that HGA causing pancytopenia is likely due to a multi-modal mechanism involving immune and non-immune platelet destruction, global bone marrow suppression, hemophagocytic lymphohistiocytosis, and myelosuppressive chemokines release.

## 4 Conclusions

A thorough history remains a key in deciding the workup and confirmation of the diagnosis. Salient features in the history such as camping, trekking, hiking in the northeastern states during spring and summer are highly suggestive of tick borne diseases and help us narrow down the differential diagnosis. A prompt diagnosis and management will facilitate an early recovery and prevent any fatal complications such as pancytopenia. This case report makes readers aware of this rare but possible presentation of anaplasma phagocytophilum induced pancytopenia, which may occur as a delayed complication, as was the case in our patient. As we continue to learn more about the pathogenesis of anaplasma infection, understanding the mechanisms helps us to better manage patients with pancytopenias and explore therapeutic interventions to reverse the cytopenias.

## Ethical approval

Obtained.

## Sources of funding

None.

## Author contribution

DS, TA, MA wrote the abstract, discussion, study concept, design, conclusion. SJ, VRN, NA, STT revised the edits, figures, chart review. HSG, VS, VJ, KR wrote the case presentation, data collection. DS, TA, AJ performed the final edit.

## Registration of research studies

1. Name of the registry: NA
2. Unique Identifying number or registration ID: NA
3. Hyperlink to your specific registration (must be publicly accessible and will be checked): NA

## Guarantor

Talal Almas.

## Provenance and peer review

Not commissioned, externally peer-reviewed.

## Consent

Written informed consent was obtained from the patient for publication of this case report and accompanying images. A copy of the written consent is available for review by the Editor-in-Chief of this journal on request.

## Declaration of competing interest

None.
